# Arabidopsis APx-R Is a Plastidial Ascorbate-Independent Peroxidase Regulated by Photomorphogenesis

**DOI:** 10.3390/antiox10010065

**Published:** 2021-01-07

**Authors:** Fernanda Lazzarotto, Khadija Wahni, Maiara Piovesana, Felipe Maraschin, Joris Messens, Marcia Margis-Pinheiro

**Affiliations:** 1 Departamento de Genética, Universidade Federal do Rio Grande do Sul, Porto Alegre 91509-900, Brazil; fernanda.lazzarotto@ufrgs.br (F.L.); maiarap@yahoo.com.br (M.P.); 2 Programa de Pós-Graduação em Biologia Celular e Molecular, Universidade Federal do Rio Grande do Sul, Porto Alegre 91509-900, Brazil; felipe.maraschin@ufrgs.br; 3 VIB-VUB Center for Structural Biology, B-1050 Brussels, Belgium; Khadija.Wahni@vub.be; 4 Brussels Center for Redox Biology, B-1050 Brussels, Belgium; 5 Structural Biology Brussels, Vrije Universiteit Brussel, B-1050 Brussels, Belgium; 6 Departamento de Botânica, Universidade Federal do Rio Grande do Sul, Porto Alegre 91509-900, Brazil

**Keywords:** ascorbate, peroxidase, photomorphogenesis, ROS

## Abstract

Peroxidases are enzymes that catalyze the reduction of hydrogen peroxide, thus minimizing cell injury and modulating signaling pathways as response to this reactive oxygen species. Using a phylogenetic approach, we previously identified a new peroxidase family composed of a small subset of ascorbate peroxidase (APx) homologs with distinguished features, which we named ascorbate peroxidase-related (APx-R). In this study, we showed that APx-R is an ascorbate-independent heme peroxidase. Despite being annotated as a cytosolic protein in public databases, transient expression of *AtAPx-R-YFP* in *Arabidopsis thaliana* protoplasts and stable overexpression in plants showed that the protein is targeted to plastids. To characterize APx-R participation in the antioxidant metabolism, we analyzed loss-of-function mutants and *AtAPx-R* overexpressing lines. Molecular analysis showed that glutathione peroxidase 7 (GPx07) is specifically induced to compensate the absence of APx-R. APx-R overexpressing lines display faster germination rates, further confirming the involvement of APx-R in seed germination. The constitutive overexpression of *AtAPx-R-YFP* unraveled the existence of a post-translational mechanism that eliminates APx-R from most tissues, in a process coordinated with photomorphogenesis. Our results show a direct role of APx-R during germinative and post-germinative development associated with etioplasts differentiation.

## 1. Introduction

Peroxidases are heme-containing enzymes present in bacteria, fungi, plants, and animals, which catalyze the reduction of hydrogen peroxide and the oxidation of a wide variety of organic and inorganic substrates [1]. Based on sequence similarity and protein structure, they are divided into the peroxidase-cyclooxygenase and the non-animal peroxidase superfamilies [2,3,4]. The latter includes twelve families of evolutionarily related enzymes found in bacteria, algae, fungi, and/or plants, which are further categorized into three large classes [2,5]. It is well-accepted that members of the non-animal peroxidase superfamily share a prokaryotic origin, and that the emergence of each family is tightly associated with processes related to mitochondria and chloroplast acquisition and countless duplication events throughout genomes’ evolution [6]. Because of their common origin, peroxidases from this superfamily share structure, folding, and catalytic sites, despite differences in their amino acid composition, presence of transit peptides, transmembrane domains, and in substrate specificity [1]. In plants, the non-animal superfamily is primarily represented by class III secretory peroxidases and class I intracellular peroxidases, which depend on ascorbate.

Ascorbate peroxidases (APx, EC.1.11.1.11) integrate the antioxidant metabolism of Archaeplastida by reducing hydrogen peroxide in the ascorbate-glutathione cycle [7,8]. Despite their specificity towards this substrate, APx can also oxidize non-physiological aromatic substrates in vitro, such as 2,2′-azinobis(3-ethylbenzothiazoline-6-sulfonic acid), pyrogallol, guaiacol, ferulic acid, and *p*-cresol, at rates comparable to ascorbate [7]. The ability to bind these molecules is given by the occurrence of two distinct binding sites, one for ascorbate and another one for aromatic molecules [9,10]. Although little is known about APx activity towards other substrates in vivo, a recent study demonstrated that APx catalyzes the hydroxylation of 4-coumarate to caffeate in lignin biosynthesis, in a reaction dependent on ascorbate and molecular oxygen [11], suggesting a role of APx in oxidizing phenolic compounds in vivo. In plants, APx activity is displayed by isoenzymes localized in distinct subcellular compartments, which are encoded by a small gene family [12]. Six genes encode APx in *A. thaliana*, which can be grouped based on their location into cytosolic (At1g07890 and At3g09640) [13], chloroplastic (At1g77490 and At4g08390) [14,15], and peroxisomal (At4g35000 and At4g35970) [16,17].

In addition to the well-described and characterized APx, we identified a subset of sequences annotated as APx homologs, which display consistent divergences when compared to other members of the APx family. One of the most striking differences is the absence of a critical residue for ascorbate binding, a highly conserved arginine at position 172 [9,18]. Moreover, phylogenetic analyses have shown that these sequences do not cluster with members of the APx family, but compose a well-supported separate family, which was named ascorbate peroxidase-related (APx-R) [19]. Later, through a comprehensive phylogenetic analysis, we were able to classify APx-R as a new class I family, along with APx, cytochrome-c peroxidase (CcP), APx-CcP hybrid, and catalase peroxidase (KatG) [20]. Recent data retrieved from basal Archaeplastida show that APx-R diverged in a chlorophyte ancestor, eventually culminating with the establishment of a distinct family of peroxidases [21]. Further, we showed that rice (*Oryza sativa* L.) APx-R is a chloroplast-targeted protein with a role in the antioxidant metabolism since the knockdown of its encoding gene induced a redox compensatory mechanism in transgenic plants. We also showed that APx-R remains as a single-copy gene in most analyzed genomes even after repeated intrachromosomic duplications involving this locus during plant genomes’ evolution [19]. The *A. thaliana* APx-R ortholog is annotated as an APx encoding gene in most databases (At4g32320; APx6) and is assumed to encode for a cytosolic protein [22]. Knockout mutants (*apx6-1*) for this gene have been functionally characterized, suggesting a role for APx-R/APx6 in oxidative protection during seed development and germination. Moreover, the loss-of-function of APx-R/APx6 led to imbalances in phytohormones, primary metabolites, and amino acids levels in mutant seeds [22].

Despite the evidence supporting APx-R classification as a distinct and yet uncharacterized peroxidase, the lack of biochemical data regarding its activity and substrate specificity hindered the unequivocal separation of APx and APx-R families. Here, we studied the purified recombinant *A. thaliana* APx-R and showed that it is a functional peroxidase that does not use ascorbate as a substrate. By transient and constitutive overexpression in plant cells, we demonstrated that APx-R is targeted to chloroplasts and undergoes a post-translational mechanism to prevent its accumulation in green tissues during plant development in a process coordinated with photomorphogenesis.

## 2. Materials and Methods

### 2.1. DNA Constructs

AtAPx-R (At4g32320) coding sequences (CDS) with and without stop codon were amplified from *Arabidopsis thaliana* leaf cDNA sample using the following primers: FOR (5′ CACCATGACGACGACGACT 3′), REV_STOP (5′ TCATAACATATTCCATTTGGCCC 3′) or REV_NOSTOP (5′ TAACATATTCCATTTGGCCC 3′). The amplified sequences were cloned into pENTR/D-TOPO (Thermo Fisher Scientific, Waltham, MA, USA) and recombined with the respective destination vector using Gateway LR Clonase II Enzyme Mix (Thermo Fisher Scientific). For protoplasts assays, APx-R CDS with and without stop codon were cloned into p2YGW7 [23] and pART7 [24] vectors, respectively. Overexpression construct was obtained through APx-R CDS cloning into pEARLEYGATE101 vector [25]. A codon optimized version of mature AtAPx-R (Figure S1) was synthesized and cloned into pET28b (Addgene) to allow the expression of a His-tagged protein in *E. coli* cells. The determination of APx-R transit peptide was based on ChloroP Prediction Server (http://www.cbs.dtu.dk/services/ChloroP/).

### 2.2. Plant Material

*A. thaliana* APx-R T-DNA insertion lines (CS851165, *apx6-1*; CS859356, *apx-r 2*) were obtained from the Arabidopsis Biological Resource Center (https://abrc.osu.edu/). Homozygous plants were identified by PCR, which followed the SIGnAL Laboratory recommendations (http://signal.salk.edu/tdnaprimers.2.html). Plants were cultivated at 22 ± 2 °C and 16 h light/8 h dark (100 µmol m^−2^ s^−1^). Overexpressing lines were obtained through floral dip transformation protocol [26]. Transgenic plants were selected on MS agar plates supplemented with B5 vitamins, 1% sucrose, and 10 mg mL^−1^ glufosinate-ammonium (Sigma-Aldrich, St. Louis, MO, USA) followed by PCR to detect the presence of the transgene. The analysis was performed on homozygous lines which were selected by resistance marker segregation analysis. Plants treated with 50 µM MG-132 (Sigma-Aldrich, St. Louis, MO, USA) or 1 µM methyl viologen dichloride hydrate (Sigma-Aldrich, St. Louis, MO, USA) were germinated on solid MS media and transferred to liquid MS for treatment; for the control sample, the vehicle was added in the same concentration. For reactive oxygen species (ROS) staining, 2′,7′-Dichlorodihydrofluorescein diacetate (H_2_DCFDA) was used. Seedlings were incubated in 10 μM H_2_DCFDA and vacuum-infiltrated for 5 min. The plant material was washed three times with double distilled water and analyzed on a fluorescent microscope.

### 2.3. Fluorescence Microscopy

Fluorescence microscopy analysis was performed on Auxio Imager D2 Microscope (Zeiss). YFP and H_2_DCFDA fluorescence were detected using MF101 Spectrum Green^TM^ filter, and chlorophyll autofluorescence using SP104v2 Cy5^TM^ filter. Confocal microscopy was performed under a FluoView 1000 confocal laser scanning microscope (Olympus, Tokyo, Japan) and images were captured with a photomultiplier tube detector.

### 2.4. Seed Germination

Seeds were sterilized in a 70% ethanol, 0.1% Triton X-100 solution for 15 min, washed several times with sterile water, and plated on MS (Murashige and Skoog) (Sigma-Aldrich, St. Louis, MO, USA) media (with B5 vitamins, 3% Phytagel™, pH 5.8) supplemented or not with 0.5 µM methyl viologen dichloride hydrate (Sigma-Aldrich). Seed germination for the expression analysis was performed on filter paper soaked on sterilized distilled water. Seeds were stratified at 4 °C in the dark for 2–3 days and incubated in a growth chamber at 22 °C in the dark or with continuous 80 μmol m^−2^ s^−1^ irradiation. Germination was defined by 1 mm radicle emergence.

### 2.5. Arabidopsis Protoplasts Assay

*A. thaliana* mesophyll protoplasts were obtained according to Wu et al. [27], and cell transformation followed protocol established by Sheen [28].

### 2.6. Quantitative Real-Time PCR (RT-qPCR)

Total RNA was extracted using the Trizol reagent (Thermo Fisher Scientific). The cDNAs were obtained by reverse transcription (RT) using Superscript II (Thermo Fisher Scientific) and a 23-polyT-V primer. Quantitative PCR was performed using the following primers: *APx-R* (At4g32320) (5′ CGGATCATGCTTTAGTGCAG 3′ and 5′ CCAAATTATTCGGGCATGTT 3′); *tAPx* (At1g77490) (5′ CGTAGCTGCAAAGTATTCTACGG 3′ and 5′ ACCACCAAAGAGAGTGGACAA 3′); *sAPx* (At4g08390) (5′ ATTACGCTGTAGCCCATGC 3′ and 5′ GTACTCGCATGAAAAGTTCTCC 3′); *APx-L* (At4g09010) (5′ TGAAACCGTTGCCTATTAATTC 3′ and 5′ CTCTGTCTCATCACAAACCAGA 3′); *PrxQ* (At3g26060) (5′ CATTGGCATTAGTGGAGATGACT 3′ and 5′ AATGCTCCAAACAGGTCTCCA); *GPx01* (At2g25080) (5′ GTCTCCGGTAACCAAAAATG 3′ and 5′ GACGAGAAAGGTTGCTGAGG 3′); and *GPx07* (At4g31870) (5′ TCGGCCCATCATTGAGATTC 3′ and 5′ CTGCAGCCCTTGCATAGAC 3′). The following primers were used as internal controls to normalize the amount of mRNA in each sample: *UBQ10* (At4g05320) (5′ GGCCTTGTATAATCCCTGATGAATAAG 3′ and 5′ ACTATGTTTCCGTTCCTGTTATCTCTTT 3′) and *PP2A* (At1g13320) (5′ TAACGTGGCCAAAATGATGC 3′ and 5′ ACCAAGCGGTTGTGGAGAAC 3′) [29]. Quantities of amplified products were compared by the 2^-DDCt^ method [30] using an Applied Biosystems StepOne Real-Time PCR System.

### 2.7. Protein Expression and Purification from E. coli

The pET28b-APx-R construct was expressed in Rosetta™(DE3) pLysS. Cells were resuspended in lysis buffer (0.1 M Hepes/NaOH pH 8.0, 0.5 M NaCl, 10% glycerol, 2 mM DTT, 20 mM Imidazole, 1 µg/mL leupeptine, 50 µg/mL DNAse, 20 mM MgCl_2_, 0.1 mg/mL AEBSF, and 1 mM ascorbate) and disintegrated in a cooled French pressure cell. The lysate was centrifuged at 18,000 rpm for 30 min, and the supernatant was incubated with Ni^2+^-Sepharose^®^ High-Performance affinity media (GE Healthcare) for 2 h at 4 °C and packed into a column. The recombinant protein was eluted as a single peak with a 400 mL linear gradient of Imidazole, and active fractions were analyzed SDS-PAGE and Western blot analysis. Purified samples were kept in 20 mM phosphate buffer (pH 8.0, 0.3 M KCl, 2 mM DTT, and 10% glycerol) and flash-frozen in liquid nitrogen after hemin reconstitution to be stored at −80 °C.

### 2.8. Hemin Reconstitution

Recombinant APx-R was incubated with 0.5 µL aliquots of a fresh 8 mg mL^−1^ hemin (Merck) in NaOH 0.1 M solution to 2 mL protein samples until the Reinheitszahl (RZ) value (A_408nm_/A_280nm_) increased to approximately 1.8. Immediately prior to use, the pH of the hemin solution was lowered to 8.5 with NaH_2_PO_4_ 1 M solution. Unbound hemin was removed by desalting chromatography.

### 2.9. Ferrous Oxidation of Xylenol Orange (FOX) Assay

To test the ability of APxR to reduce H_2_O_2_, the FOX assay was performed as previously described [31,32]. Briefly, the reaction mixture contained APxR (0.5 µM or 5 µM) and 250 µM H_2_O_2_ in 50 mM Tris/HCl pH 8, 0.3 M KCl. Afterward we incubated the mixtures at 2 different temperatures: room temperature or 36 °C. At different time points, 10 µL of this reaction mix were mixed with 490 µL of FOX reagent (100 µM Xylenol orange, 250 µM ammonium ferrous (II) sulfate, 100 mM sorbitol, and 25 mM H_2_SO_4_). After incubation for 30 min in the dark, 200 µL was pipetted into a clear-bottom 96-well plate (add brand of the plate). The absorbance at 560 nm was measured using a SpectraMax 340PC plate reader (Molecular devices). Prior to the FOX reaction, a buffer exchange for APxR was performed to 20 mM Tris/HCl pH 8, 0.3 M KCl using Biospin columns (Bio-rad, Hercules, CA, USA) to remove excess of DTT. The final concentrations of the controls in the reaction mixture are: 1.5 mg/mL BSA (Sigma-Aldrich) and 15 units catalase (Sigma-Aldrich).

### 2.10. Enzymatic Assays

Peroxidase activity was assayed spectrophotometrically based on Shigeoka et al. [33]. The assay mixture was kept at 36 °C and contained 100 mM potassium phosphate buffer at pH 6, 6.5, or 7; 0.1 mM H_2_O_2_; the analyzed substrate; and the enzyme. Ascorbic acid (0.4 mM), pyrogallol (20 mM) and guaiacol (10 mM) were evaluated as potential electron donors following a decrease in the absorbance at 290 nm, or an increase in the absorbance at 430 and 470 nm, respectively.

## 3. Results

### 3.1. APx-R Is a Peroxidase that Does not Use Ascorbate as Substrate

**Figure 1:**
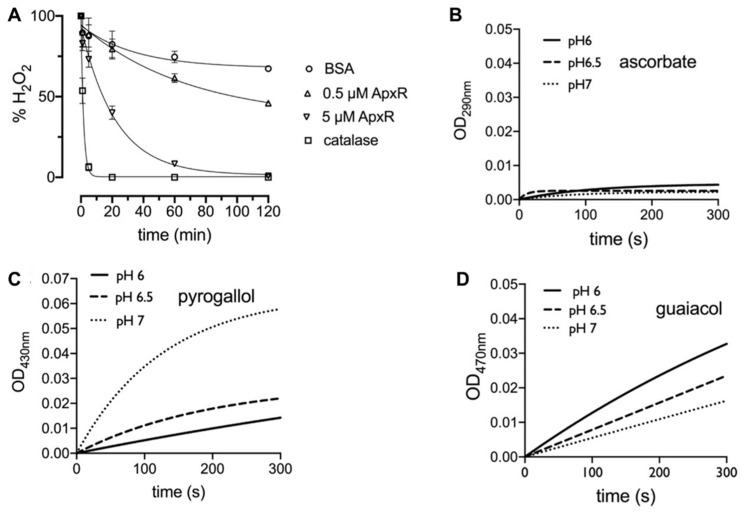
APx-R displays heme peroxidase activity. (**A**) FOX assay showing H_2_O_2_ reduction rate in the presence of BSA (negative control), APx-R at 0.5 and 5 µM, and catalase (positive control); and (B–D) the oxidation of the electron donors L-ascorbic acid (**B**), pyrogallol (1 µM) (**C**), and guaiacol (0.2 µM) (**D**) mediated by APx-R (1 µM) was analyzed at pH 6, 6.5, and 7 in the presence of 0.1 mM H_2_O_2_ as substrate. The oxidation products of the analyzed substrates were monitored at 290, 430, and 470 nm, respectively. The exponential fitting of the mean of three independent measurements is shown. The reaction with 1 µM APxR for guaiacol was too fast to monitor and was decreased to 0.2 µM.

Despite the strong evidence supporting APx-R classification as a family of peroxidases that does not use ascorbate as substrate [4,20], proteins belonging to this family remain currently categorized as APx homologs in most databases and the literature. To investigate APx-R activity, we expressed His-tagged *A. thaliana* APx-R in *E. coli*. The recombinant protein was purified from bacterial lysate on a Ni^2+^-Sepharose column, and elution fractions were analyzed on SDS-PAGE gel and Western blot (Figure S2). After hemin reconstitution, the spectrophotometric determination of the Reinheitszahl (RZ) value (A403 nm/A280 nm) of the purified samples shifted from 0.05 to 0.9, indicating protein binding to the heme. Consumption of hydrogen peroxide by APx-R was measured using the ferrous iron-catalyzed oxidation of xylenol orange (FOX assay) using distinct electron donors (Figure 1A). We evaluated APx-R reaction with ascorbate, pyrogallol, and guaiacol, in the presence of hydrogen peroxide. As for other heme peroxidases, recombinant APx-R effectively reduced H_2_O_2_ in the presence of pyrogallol and guaiacol in vitro (Figure 1C,D); on the other hand, no reaction with ascorbate was detected (Figure 1B).

### 3.2. Arabidopsis APx-R Is a Chloroplast-Targeted Enzyme

**Figure 2:**
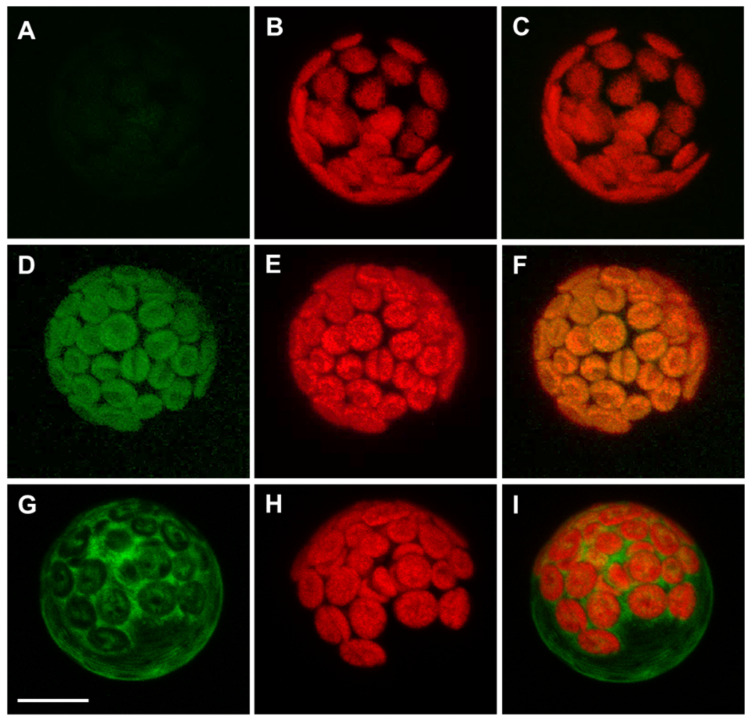
A. thaliana APx-R-YFP is targeted to chloroplasts. Confocal microscope images of transient expression assay performed on A. thaliana mesophyll protoplasts: (**A**–**C**) non-transformed cells; (**D**–**F**) expression of a carboxi-terminal YFP fusion; and (**G**–**I**) expression of amino-terminal YFP fusion. (**A**,**D**,**G**) YFP fluorescence; (**B**,**E**,**H**) chlorophyll autofluorescence; and (**C**,**F**,**I**) merge. Scale: 8 µm

Previous data from our group show that APx-R is targeted to the chloroplasts in rice [19]. *A. thaliana* APx-R, however, is believed to be a cytosolic protein [22], suggesting that it might present distinct features within this species. Similar to the thylakoid and stromal APxs, APx-R also harbors an N-terminal extension that contains a putative transit peptide (Figure S3). To determine its functionality, we performed a transient expression assay in *A. thaliana* mesophyll protoplasts. An YFP tag was fused to either the N- or C-terminal end of APx-R under the control of a constitutive promoter. Whereas the C-terminal fusion protein (APx-R-YFP) was targeted to the chloroplasts (Figure 2D–F), the N-terminal fusion (YFP-APx-R) was retained in the cytoplasm (Figure 2G–I), suggesting the N-terminal tag hindered the recognition of the plastidial transit peptide. Public expression data suggest that AtAPx-R transcripts are prominently expressed in mature seeds [34,35].

**Figure 3:**
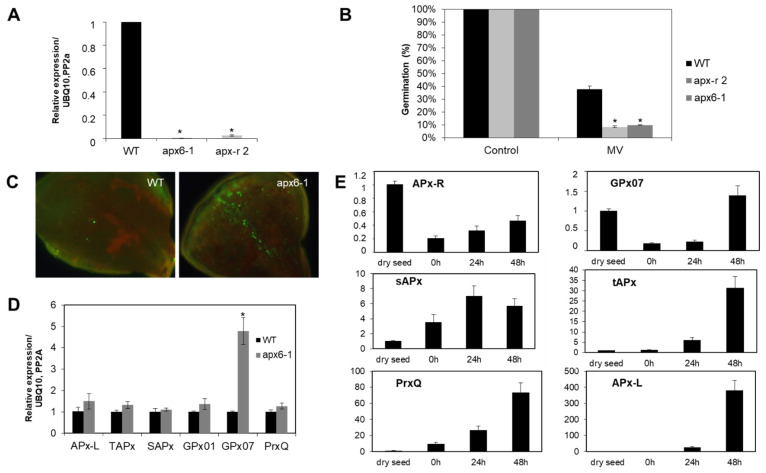
APx-R participates on chloroplast antioxidant defense. (**A**) Relative expression of AtAPx-R (At4g32320) gene on 10-day-old wild-type, apx6-1 (CS851165) and apx-r 2 (CS859356) seedlings. (**B**) Average germination rate of apx6-1, apx-r 2, and wild-type seeds in the presence of 0.5 µM methyl-viologen. Values are the mean of three biological replicates (*n* = 50). (**C**) Ten-day-old apx6-1 and wild-type seedlings were exposed to 1 µM methyl-viologen for 12 h and reactive oxygen species (ROS) accumulation was analyzed after incubation with the fluorescent probe 2′,7′-Dichlorodihydrofluorescein diacetate (DCFDA). (**D**) RT-qPCR analysis performed on 10-day-old plants shows the upregulation of glutathione peroxidase 7 (GPx07), whereas other chloroplast peroxidases encoding genes were not affected in apx6-1. (**E**) Relative expression of chloroplast peroxidases throughout germination in wild-type Col-0 seeds evaluated by RT-qPCR. Four time points were analyzed: dry seed, 0 h (after stratification), and 24 and 48 h into photoperiodic illumination. All gene expression analyses were normalized by two reference genes: PP2A and UBQ10. Each time point consisted of three biological replicates and four technical replicates. At least two independent experiments were performed. * Significant difference according to Student’s *t*-test (*p* < 0.05).

In addition, analysis carried out with loss-of-function mutants showed that APx-R is involved in seed protection during germination [22]. Loss-of-function mutant lines (apx6-1; apx-r 2) exhibited a 73% reduction on the germination rate in the presence of the chloroplast stress agent methyl viologen (Figure 3A,B), which also prompted knockout seedlings to accumulate reactive oxygen species (ROS) in the cotyledons (Figure 3C). To assess how the chloroplast antioxidant metabolism is compensating for the lack of APx-R in a loss-of-function mutant, we analyzed the expression of other peroxidases targeted to the same subcellular compartment. Quantitative PCR showed that glutathione peroxidase 7 (GPx07) encoding gene is induced more than four-fold in apx6-1 plants, while APx encoding genes showed no modulation (Figure 3D). The expression profile of chloroplastic peroxidases throughout germination showed that AtAPx-R and AtGPx07 have identical regulatory function, with high transcripts abundancy in dry seeds followed by drastic repression after stratification, in contrast to what was observed for peroxiredoxin Q (Prx-Q), tAPx, sAPx, and APx-L (Figure 3E).

**Figure 4:**
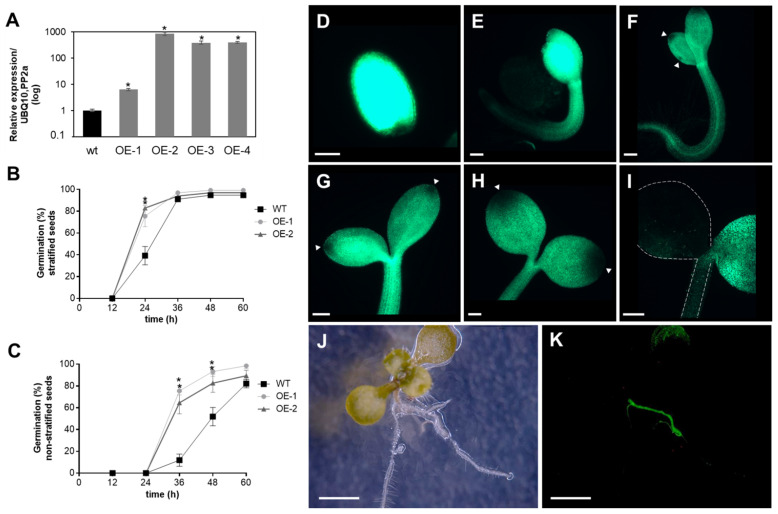
Analysis of AtAPx-R-YFP overexpressing plants. (**A**) Relative expression of AtAPx-R-YFP OE lines. Two-week-old seedlings from wild-type Col-0 and four independent transgenic lines were analyzed by RT-qPCR. Gene expression analysis was normalized by two reference genes: PP2A (At1g13320) and UBQ10 (At4g05320). Each time point consisted of three biological replicates (*n* = 5) and four technical replicates. (**B**) Time-course germination of stratified and (**C**) non-stratified seeds of two representative AtAPx-R-YFP OE lines and wild-type Col-0. Each time point consisted of three biological replicates (*n* = 45). Two independent experiments were performed. (**D**–**I**) Seeds from AtAPx-R-YFP OE-2 line were germinated in light and analyzed throughout early stages of plant development. Arrowheads indicate early loss of APx-R-YFP fluorescence. Dashed lines were added for plant outlines determination. (**J**,**K**) Representative 10-days-old plant from AtAPx-R-YFP OE-1 line analyzed under a stereomicroscope. (**J**) Bright field; and (**D**–**I**,**K**) YFP fluorescence. * Significant difference according to Student’s *t*-test (*p* < 0.05). Scale (**D**) 400 µm; (**E**–**I**) 200 µm; and (**J**,**K**) 1 mm.

Previously, we showed that APx-R is maintained as a single-copy gene in most plant genomes, even after repeated genomic duplications involving the chromosomic region that contains this gene [19]. To investigate whether high expression levels of APx-R could lead to a deleterious phenotype, plants overexpressing APx-R-YFP were produced (Figure 4A). The overexpression of APx-R prompted seeds to germinate more quickly, a phenotype that was more pronounced when seeds were not subjected to stratification (Figure 4B,C). Despite the observed phenotype, transgenic seeds did not show significant differences in size, shape, or color (Figure S4). Fluorescence microscopy analysis confirmed the accumulation of recombinant APx-R-YFP in the transgenic plants. However, analysis carried out at early stages of plant development showed that constitutively expressed APx-R-YFP is degraded in seedlings, via a process initiated at the periphery of the cotyledons (Figure 4D–I). After the emergence of the first pair of true leaves, APx-R-YFP can only be detected at significant levels in roots (Figure 4J,K) and guard cells (Figure S5), regardless of the expression level of the transgene (Figure S6). This phenotype is consistently observed in over ten independent lines with no significant difference among them.

### 3.3. Degradation of Recombinant APx-R Is Coordinated with De-Etiolation

**Figure 5:**
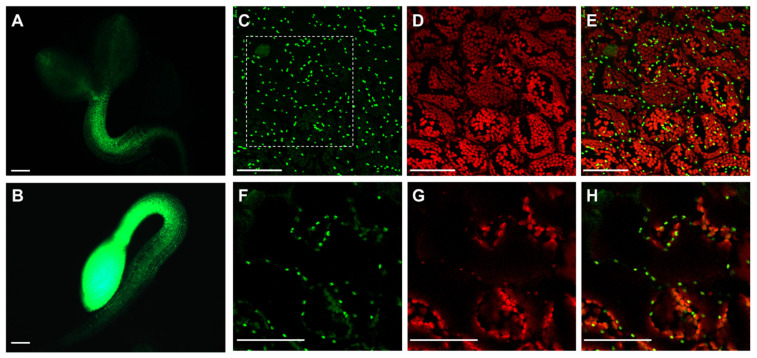
Light reduces APx-R-YFP signal in APx-R-YFP OE seedlings. Fluorescence microscopy analysis of two-day-old plants from AtAPx-R-YFP OE-1 line germinated in the light (**A**) or dark (**B**). (**C**–**E**) Confocal laser scanning microscope z-stack images from residual fluorescence localized on cotyledons of a plant germinated in the light under a 60× objective. (**F**–**H**). Zoom-in cross-section image from the dashed square indicated on (**C**). YFP fluorescence is shown in green (**A**–**C**,**F**) and chlorophyll autofluorescence (**D**,**E**,**G**,**H**), in red. Scale (**A**,**B**) 200 µm; and (**C**–**H**) 50 µm.

Because of the contrasting phenotype observed in shoots and roots concerning APx-R-YFP accumulation, we speculated that APx-R degradation could be restricted to chloroplasts. To test this hypothesis, APx-R-overexpressing seeds were germinated in the presence or absence of light. While photomorphogenic seedlings lost most of the YFP fluorescence in green tissues within two days, seedlings germinated in the dark exhibited high levels of fluorescence throughout the whole plant (Figure 5A,B). APx-R degradation after light exposure seems to be an irreversible process since protein accumulation could not be restored after plants were transferred to the dark for up to five days (data not shown). Confocal microscopy analysis of residual fluorescence in four-day-old seedlings grown under light showed that APx-R-YFP is not localized in the stroma, but in small vesicles present inside and outside the chloroplasts (Figure 5C–H).

**Figure 6:**
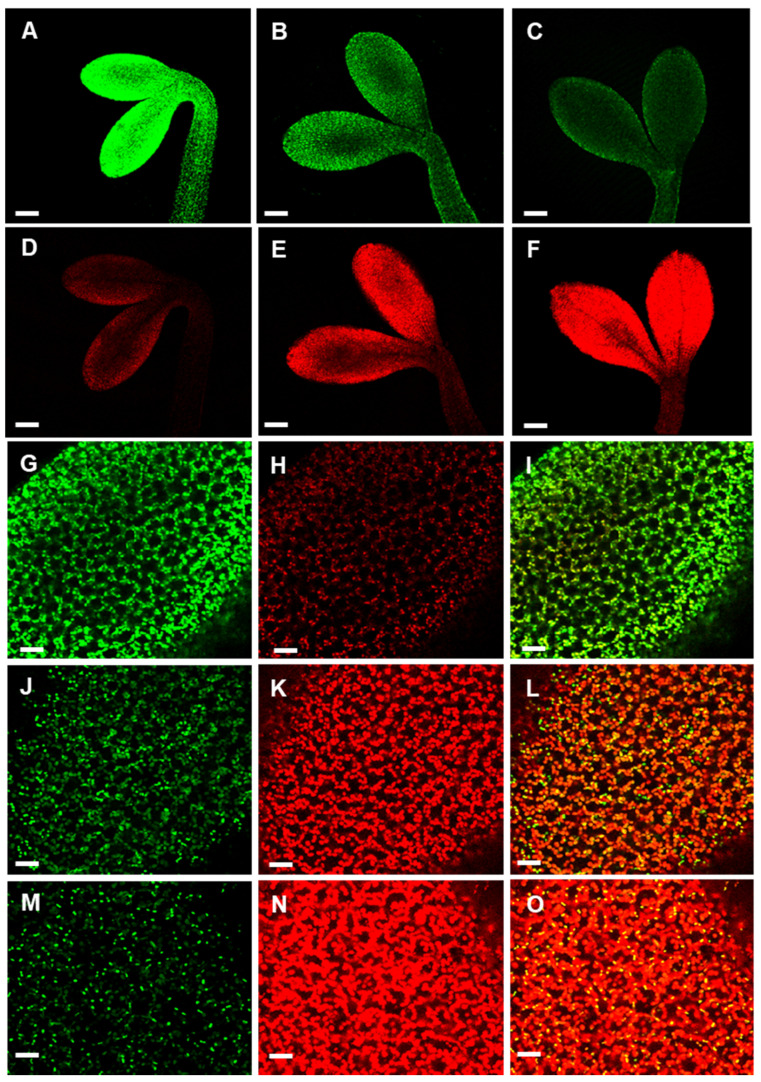
Time-course of light-induced APx-R-YFP degradation. Confocal z-stack images from two-day-old At APx-R-YFP OE seedlings germinated in the dark and exposed to light for: 0 h (**A**,**D**,**G**–**I**); 2 h (**B**,**E**); 6 h (**C**,**F**,**J**–**L**); and 20 h (**M**–**O**). Magnification (**A**–**E**) 20× objective; and (**G**–**O**) 60× objective. APx-R-YFP fluorescence is shown in green (**A**–**C**,**G**,**I**,**J**,**L**,**M**,**O**); and chlorophyll autofluorescence in red (**D**–**F**,**H**,**I**,**K**,**L**,**N**,**O**). Scale: (**A**–**F**) 200 µm; and (**G**–**O**) 30 µm.

The occurrence of chlorophyll in these vesicles, associated with their morphology and distribution inside the cells, suggests that APx-R might be localized in plastoglobuli [25,26]. Experimental data from proteomic analyses deposited in The Plant Proteome Database (http://ppdb.tc.cornell.edu/) confirm APx-R occurrence in stroma and plastoglobuli, as well as in thylakoid membranes, which are also implicated in plastoglobuli formation (Table S1). To assess whether this localization is correlated to APx-R degradation, we analyzed seedlings germinated in the dark for two days after exposure to 0, 2, 6, and 20 h of light. Confocal microscopy analyses showed that APx-R-YFP is rapidly degraded in a process coordinated with chlorophyll accumulation (Figure 6A–F). While APx-R subcellular localization is remarkably stromal at 0 h of light exposure (Figure 6G–I), the continued presence of light triggered its relocation, in a process which culminated with protein degradation (Figure 6J–O).

### 3.4. APx-R Degradation Is Sustained during Plant Development

**Figure 7:**
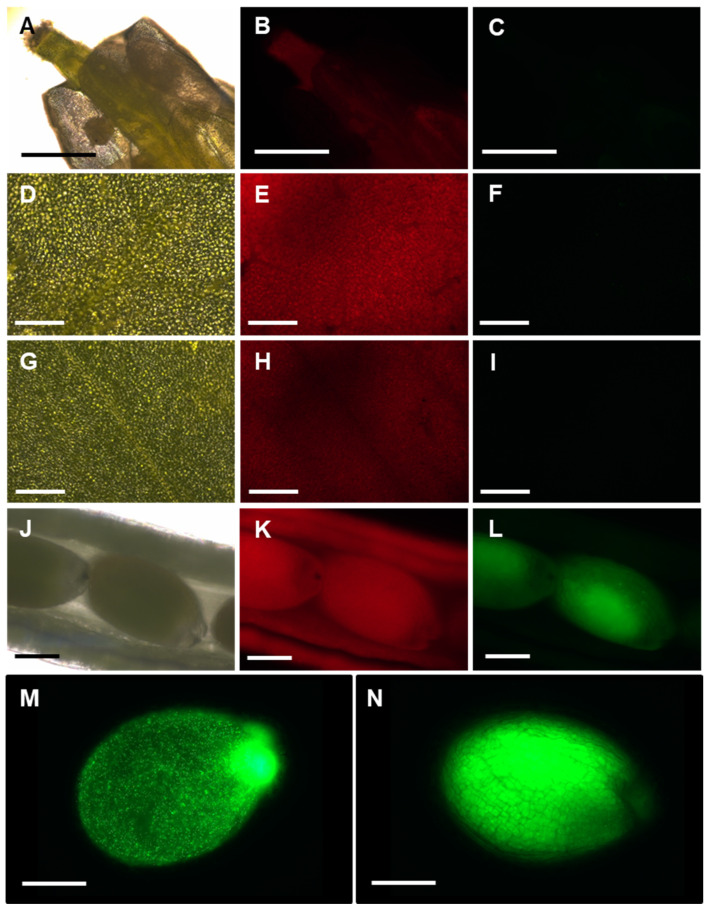
APx-R-YFP accumulation in vegetative and reproductive tissues. Fluorescence microscopy images of a representative AtAPx-R-YFP OE-2: flower (**A**–**C**); rosette leaf (**D**–**F**); cauline leaf (**G**–**I**); immature seeds (**J**–**M**); and mature seed (**N**). Bright field (**A**,**D**,**G**,**J**); chlorophyll autofluorescence (**B**,**E**,**H**,**K**); and YFP fluorescence (**C**,**F**,**I**,**L**–**N**). Scale: (**A**–**C**) 1 mm; (**D**–**I**) 100 µm; and (**J**–**N**) 400 µm.

To further understand in which tissues APx-R accumulation is regulated at the post-translational level, mature plants from transgenic lines were analyzed with fluorescence microscopy. This analysis showed that APx-R-YFP does not significantly accumulate in flowers (Figure 7A–C), rosette leaves (Figure 7D–F), and inflorescence leaves (Figure 7G–I). However, in developing seeds, APx-R-YFP was detected at considerably higher levels (Figure 7J–N). Note that this accumulation was not homogeneous throughout all stages of seed development. Treatment of AtAPx-R-YFP OE seedlings with MG-132 showed that APx-R degradation is a proteasome independent process (Figure S7).

## 4. Discussion

Ascorbate peroxidases are considered one of the key components of hydrogen peroxide enzymatic removal in plants. Despite the overall similarity observed for APx and APx-R, phylogenetic analyses have shown a clear separation between both families [4,19,20], highlighting the existence of a distinct and yet uncharacterized peroxidase in plants antioxidant metabolism. Not only APx and APx-R belong to the same superfamily of peroxidases, but recent evidence from basal Archaeplastida indicates that APx-R diverged from APx in a chlorophyte ancestor, suggesting a strong evolutionary basis for its overall homology [21]. Members of the APx-R family present a highly conserved gene structure and consistent divergences in amino acid composition compared to APx [19]. A striking difference between both families lies in the occurrence of an ascorbate binding site, an arginine at position 172 that in APx is involved in the interaction with its electron donor [9,18]. The loss of Arg172 in the APx-R family prompted us to hypothesize that this peroxidase should not be able to use ascorbate as substrate, an assumption which has now been confirmed. Similar to other heme peroxidases, APx-R effectively binds the heme and oxidizes pyrogallol and guaiacol while reducing hydrogen peroxide in vitro, showing that this protein retained its function as heme peroxidase. Nevertheless, APx-R must have evolved towards the acquisition of distinct functions or the ability to oxidize different electron donors. Determining the exact electron donor, which will form the basis for accurate classification of this enzyme, is beyond the scope of this study.

Our results show that *A. thaliana* APx-R is a chloroplast targeted protein subjected to an intricate regulatory mechanism, which prevents APx-R from accumulating in most tissues. The photomorphogenic development acts as a trigger of a post-translational mechanism that leads to APx-R translocation from stroma to small vesicles, culminating in its degradation and, hence, drastic reduction of APx-R-YFP signal in transgenic plants as a consequence. Based on our imaging data, it is not clear whether these small microcompartments are indeed plastoglobuli. However, identical fluorescence pattern has been shown for the plastoglobuli-specific fibrillin 1B [36]. In addition, it has been shown previously by proteomic data that APx-R is located in both stroma and plastoglobuli [37,38,39,40], which suggests that APx-R might translocate between these compartments. This phenomenon has been observed for all the transgenic lines, regardless of the expression level of the transgene. Plastoglobuli exhibit a narrow proteome, and, while most proteins seem to be specific and exclusively located in these microcompartments, some are thought to be recruited under specific conditions. Plastoglobuli have been implicated in plastids transition associated with de-etiolation and senescence, and even in plant responses to abiotic stresses [41]. Studies showed that they can be expelled from chloroplasts into the cytoplasm, to be digested, e.g., in the vacuole [41]. Our confocal analyses have shown the occurrence of APx-R-YFP-containing vesicles inside and outside chloroplasts. In addition, we showed that APx-R degradation is a proteasome-independent process, supporting the hypothesis of vacuolar degradation of APx-R.

Previous analysis performed in loss-of-function mutants [22] and now in overexpressing lines have shown a consistent role for APx-R in seed antioxidant metabolism. In addition to the altered germination rates verified for both genotypes, the knockout of *APx-R* modulated the levels of phytohormones and other metabolites in the seeds [22]. Interestingly, analyses performed in overexpressing lines have shown that APx-R-YFP can accumulate in seeds and at early stages of development, supporting a primary role for this peroxidase in seed metabolism and germination. High levels of APx-R-YFP have also been observed in plant roots and stomata, suggesting the type of plastid, tissue, and/or developmental context might display a key role in APx-R stability.

For many years, antioxidant enzymes mostly have been associated with cell protection; therefore, it seemed plausible to cope with the idea that, the more antioxidant enzymes, the better. This does not seem to be the case for APx-R. We have shown that APx-R is encoded by a single gene in virtually all plant genomes, apart from the gene families observed for APx [19]. This configuration has been maintained throughout genomes’ evolution even after repeated intrachromosomic duplications involving *APx-R* loci. Our analyses showed that the duplicated *APx-R* genes were rapidly lost, even after recent duplication events marked by a large extent of sinteny between both genomic regions. Taken together, our results suggest that plants do not benefit from high levels of APx-R, at least in some tissues. In future studies, the biochemical characterization of this enzyme and its endogenous electron donor will form the basis to fully understand the regulatory mechanism underlying its peroxidase functionality in antioxidant metabolism and plant development.

## 5. Conclusions

We showed that APx-R is an ascorbate-independent and tightly regulated peroxidase. Plastid maturation triggered by light promotes APx-R translocation from stroma to plastoglobuli, ultimately leading to APx-R degradation through a proteasome-independent mechanism. The results here presented unequivocally distinguish APx-R from the other APx family members and show that this distinct peroxidase accumulates in seeds, further confirming its prominent role in seed redox homeostasis.

## Data Availability

Data is contained within the article or supplementary material.

## Supplementary Materials

The following are available online at https://www.mdpi.com/2076-3921/10/1/65/s1, Figure S1: Coding sequence synthesized to express recombinant APx-R in *E. coli*, Figure S2: Overexpression and purification of recombinant APx-R in *E. coli*, Figure S3: Protein alignment of Arabidopsis APx-R and APx proteins, Figure S4: Morphologic analysis of apx-r and AtAPx-R-YFP OE seeds, Figure S5: APx-R-YFP signal is retained in guard cells chloroplasts, Figure S6: Recombinant AtAPx-R-YFP is detectable in roots but does not accumulate in green tissues, Figure S7: APx-R degradation is a proteasome-independent process, Table S1: Identification of Arabidopsis APx-R in proteomic data deposited on The Plant Proteome Database (http://ppdb.tc.cornell.edu/).
