# Supplementary material for: Arabidopsis APx-R Is a Plastidial Ascorbate-Independent Peroxidase Regulated by Photomorphogenesis
Source: Antioxidants (Basel). 2021 Jan 7;10(1):65. doi: 10.3390/antiox10010065 (PMC7825652; doi:10.3390/antiox10010065)
Supplement: Supplementary file 1 [file antioxidants-10-00065-s001.pdf]

ATGGGTCATCACCATCACCATCACGGCGGTGAGAACCTGTACTTCCAATCCAATGGCAGCAGCCGTGCAT
GGCGCATTCGCTGCCTGAGCGATGATCCGGGTAGCAGCCATGTGTTTGTGGCGAGCCGTCGCAAAATGGT
GGTGCTGCTGAGCACCGTGCAGCTGCTGAGCCATATGCTGCCGCAGAACGGCAACGCGGCGGAAATTTAT
CCGGTGATGCAGAACGAAATTCGCAAAGTGGTGACCAAAGGCAAAGCGGCGGGCGTGCTGCGCCTGGTGT
TTCATGATGCGGGCACCTTTGAACTGGATGATCATAGCGGTGGCATTAACGGTAGCATTGCGTATGAACT
GGAACGTCCGGAAAACATTGGCCTGAAGAAAGCCTGAAAGTGCTGGCGAAAGCGAAAGTGAAAGTGGAT
GAAATTCAGCCGGTGAGCTGGGCGGATATGATTAGCGTGGCAGGCAGCGAAGCGGTGAGCATTTGCGGTG
GTCCGACCATTCCGGTGGTGCTGGGTCGTCTGGATAGCGCGCAGCCGGATCCGGAAGGCAAACTGCCGCC
GGAAACCCTGAGCGCGAGCGGCCTGAAAGAATGCTTTAAACGCAAAGGCTTTAGCACCCAGGAACTGGTG
GCGCTGAGCGGCGCGCATACCATTGGCAGCAAAGGCTTTGGCGATCCGACCGTGTTTGATAACGCGTATT
ATAAAATTCTGCTGGAAAAACCGTGGACCAGCACCAGCAAAATGACCAGCATGGTGGGCCTGCCGAGCGA
TCATGCGCTGGTGCAGGATGATGAATGCCTGCGCTGGGTGAAACGCTATGCGGAAGATCAGGATAAGTTC
TTTGAAGATTTCACCAACGCGTATATCAAACTGGTGAACAGCGGTGCGAAATGGAACATGCTGTGA

Figure S1. Coding sequence synthetized to express recombinant APx-R in *E. coli*. A fusion protein was produced using pET28b vector (Addgene). Bases highlighted in yellow encode a 6xHis-tag and in green, a cleavage site for TEV protease. In gray, codon optimized mature APx-R CDS (which excluded bases encoding the plastid transit peptide, determined by ChloroP Prediction Server - http://www.cbs.dtu.dk/services/ChloroP/).

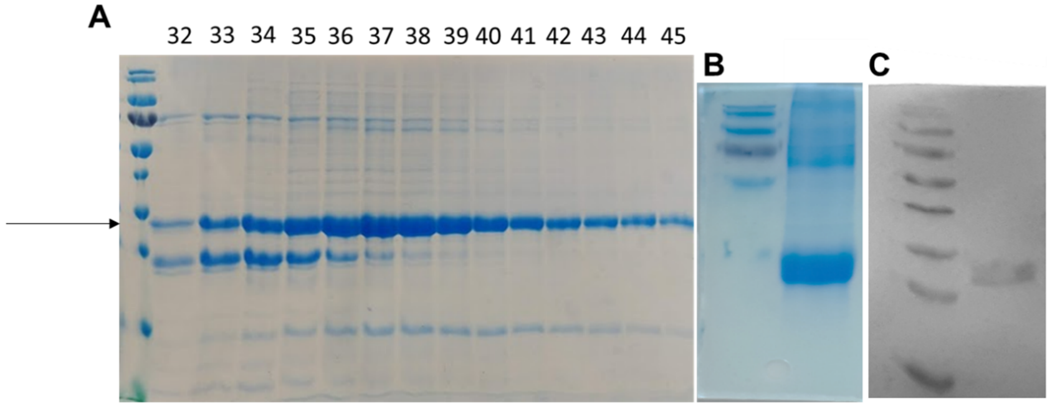

Figure S2. Overexpression and purification of recombinant APx-R in E. coli. A) SDS-PAGE analysis of elution fractions 32 to 45. The indicated protein (arrow) correlates in size with the calculated molecular mass of 33 kDa obtained from the deduced amino acid sequence of the cloned construct. Samples 38-50 were combined and analyzed by SDS-PAGE (B) and western blot (C), using anti-His antibody.

```
                 *        20         *        40         *        60         *
APx-R : -MTTTTASLVKTFLFRCDSFSSFKFKCKFESPAKTRLLSPATEKH------VVRSSRAWRIRCLSDDPGSSHVFVAS :  70
APx01 : ----------------------------------------------------------------------------- :   -
APx02 : ----------------------------------------------------------------------------- :   -
APx03 : ----------------------------------------------------------------------------- :   -
APx05 : ----------------------------------------------------------------------------- :   -
SAPx  : MAERVSLTLNGTLLSPPPTTTTTTMSSSLRSTTAASLLLRSSSSSSRSTLTLSASSSLSFVRSLVSSPRLSSSSSLS :  77
TAPx  : --MSVSLSAASHLLC---------------SSTRVSLSPAVTSSSSSPVVALSSSTSPHSLGSVASSSLFPHSSFVL :  60

         80         *       100         *       120         *       140         *
APx-R : RRKMVVLLSTVQLLSHMLPQNGNAAEIYPVM--QNEIRKVVTKGKAAGVL-RLVFHDAGTFELDDHS----GGINGS : 140
APx01 : --------------MTKNYPTVSEDYKKAVEKCRRKLRGLIAEKNCAPIMVRLAWHSAGTFDCQSRT----GGPFGT :  59
APx02 : -------------MVKKSYPEVKEEYKKAVQRCKRKLRGLIAEKHCAPIVLRLAWHSAGTFDVKTKT----GGPFGT :  60
APx03 : ----------------MAAPIVDAEYLKEITKARRELRSLIANKNCAPIMLRLAWHDAGTYDAQSKT----GGPNGS :  57
APx05 : -----------------MAVNVDAEYLKEIEKTRRDLRALISSRNCAPIMLRLAWHDAGTYDAKKKT----GGANGS :  56
SAPx  : QKKYRIASVNRSFNSTTAATKSSSSDPDQLKNAREDIKELLSTKFCHPILVRLGWHDAGTYNKNIKEWPQRGGANGS : 154
TAPx  : QKKHPINGT----STRMISPKCAASDAAQLISAKEDIKVLLRTKFCHPILVRLGWHDAGTYNKNIEEWPLRGGANGS : 133
                                              l     c pi  RL wH AGT           GG  G

           160         *       180         *       200         *       220         *
APx-R : IAY--ELERPENIGLKKSLKVLAKAKVKVDEIQPVSWADMISVAGSEAVSICGGPTIPVVLGRLDSA---QPDPEGK : 212
APx01 : MRFDAEQAHGANSGIHIALRLLDPIR---EQFPTISFADFHQLAGVVAVEVTGGPDIPFHPGREDKP---QPPPEGR : 130
APx02 : IRHPQELAHDANNGLDIAVRLLDPIK---ELFPILSYADFYQLAGVVAVEITGGPEIPFHPGRLDKV---EPPPEGR : 131
APx03 : IRNEEEHTHGANSGLKIALDLCEGVK---AKHPKITYADLYQLAGVVAVEVTGGPDIVFVPGRKDSN---VCPKEGR : 128
APx05 : IRFKEELNRPHNKGLEKAVAFCEEVK---AKHPRVSYADLYQLAGVVAVEVTGGPAIPFTPGRKDA----DSADDGE : 126
SAPx  : LRFDIELKHAANAGLVNALNLIKDIK---EKYSGISYADLFQLASATAIEEAGGPKIPMKYGRVDASGPEDCPEEGR : 228
TAPx  : LRFEAELKHAANAGLLNALKLIQPLK---DKYPNISYADLFQLASATAIEEAGGPDIPMKYGRVDVVAPEQCPEEGR : 207
         r   E     N Gl  a       k         s AD  qlA   A e  GGP Ip   GR D         eG

              240         *       260         *       280         *       300
APx-R : LPP--ETLSASGLKECFKR-KGFSTQELVALSGAHTIG-----SKGF---------------------GDPTVFDNA : 260
APx01 : LPD--ATKGCDHLRDVFAKQMGLSDKDIVALSGAHTLGRCHKDRSGF---------EGA-------WTSNPLIFDNS : 189
APx02 : LPQ--ATKGVDHLRDVFGR-MGLNDKDIVALSGGHTLGRCHKERSGF---------EGA-------WTPNPLIFDNS : 189
APx03 : LPD--AKQGFQHLRDVFYR-MGLSDKDIVALSGGHTLGRAHPERSGF---------DGP-------WTQEPLKFDNS : 186
APx05 : LPN--PNEGASHLRTLFSR-MGLLDRDIVALSGGHTLGRAHKERSDF---------EGP-------WTQDPLKFDNS : 184
SAPx  : LPDAGPPSPATHLREVFYR-MGLDDKDIVALSGAHTLGRSRPERSGWGKPETKYTKEGPGAPGGQSWTPEWLKFDNS : 304
TAPx  : LPDAGPPSPADHLRDVFYR-MGLDDKEIVALSGAHTLGRARPDRSGWGKPETKYTKTGPGEAGGQSWTVKWLKFDNS : 283
        LP         hLr  F r mGl d  iVALSG HTlGr    rsg           g        wt   l FDNs

         *       320         *       340         *       360         *       380
APx-R : YYKILLEKPWTSTSKMTSMVGLPSDHALVQDDECLRWVKRYAEDQDKFFEDFTNAYIKLVNSGAKWNML-------- : 329
APx01 : YFKELLSG------EKEGLLQLVSDKALLDDPVFRPLVEKYAADEDAFFADYAEAHMKLSELG--FADA-------- : 250
APx02 : YFKEILSG------EKEGLLQLPTDKALLDDPLFLPFVEKYAADEDAFFEDYTEAHLKLSELG--FADKE------- : 251
APx03 : YFVELLKG------ESEGLLKLPTDKTLLEDPEFRRLVELYAKDEDAFFRDYAESHKKLSELG--FNPNSSAGK--- : 252
APx05 : YFVELLKG------ETPGLLQLKTDKALLDDPKFHPFVKLYAKDEDMFFKAYAISHKKLSELG--FNPPRRIPS--- : 250
SAPx  : YFKEIKEK------RDEDLLVLPTDAAIFEDSSFKVYAEKYAADQDAFFKDYAVAHAKLSNLGAEFNPPEGIVI--- : 372
TAPx  : YFKDIKEK------RDDDLLVLPTDAALFEDPSFKNYAEKYAEDVAAFFKDYAEAHAKLSNLGAKFDPPEGIVIENV : 354
        Yf                ll L  D al  D  f      YA D d FF dy   h KLs lG  f

            *       400         *       420         *       440         *
APx-R : ------------------------------------------------------------------------ :   -
APx01 : ------------------------------------------------------------------------ :   -
APx02 : ------------------------------------------------------------------------ :   -
APx03 : --------------------------------AVADSTILAQSAFGVAVAAAVVAFGYFYEIRKRMK----- : 287
APx05 : --------------------------------AVTQQTL------GIAVAAAVVIFTICYEASRRGK----- : 279
SAPx  : ------------------------------------------------------------------------ :   -
TAPx  : PEKFVAAKYSTGKKELSDSMKKKIRAEYEAIGGSPDKPLPTNYFLNIIIAIGVLVLLSTLFGGNNNSDFSGF : 426
```

Figure S3. Protein alignment of Arabidopsis APx-R and APx proteins. Catalytic residues are marked in blue and ascorbate-binding arginine, in green. APx-R chloroplast transit peptide is indicated in orange, according to ChloroP Prediction Server (http://www.cbs.dtu.dk/services/ChloroP/) (APx-R, At4g32320; APx1, At1g07890; APx2, At3g09640; APx3, At4g35000; APx5, At4g35970; TAPx, At1g77490; SAPx, At4g08390).

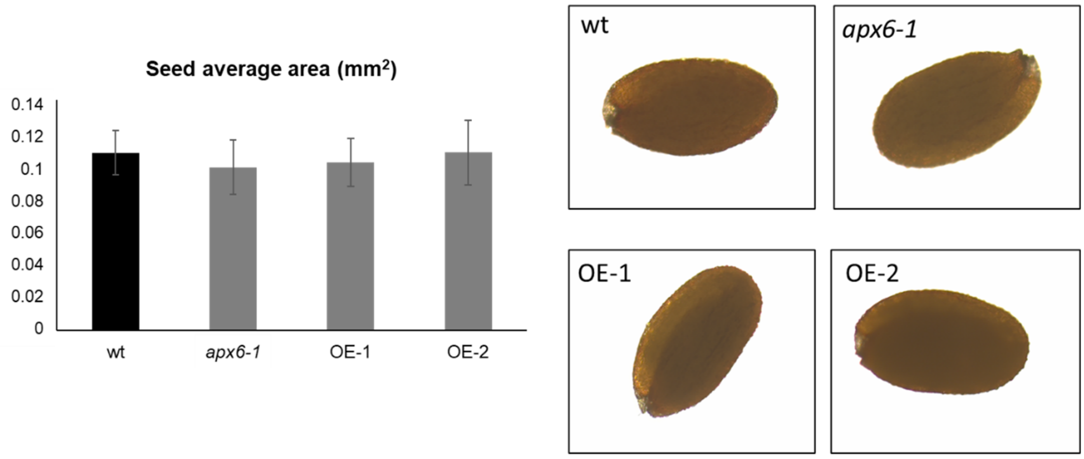

Figure S4. Morphologic analysis of apx-r and AtAPx-R-YFP OE seeds. Thirty seeds from each genotype (Col-0 WT, *apx6-1*, AtAPx-R-YFP OE-1, and OE-2) were photographed and measured on ImageJ software. No significant differences were observed for seed average area and overall morphology.

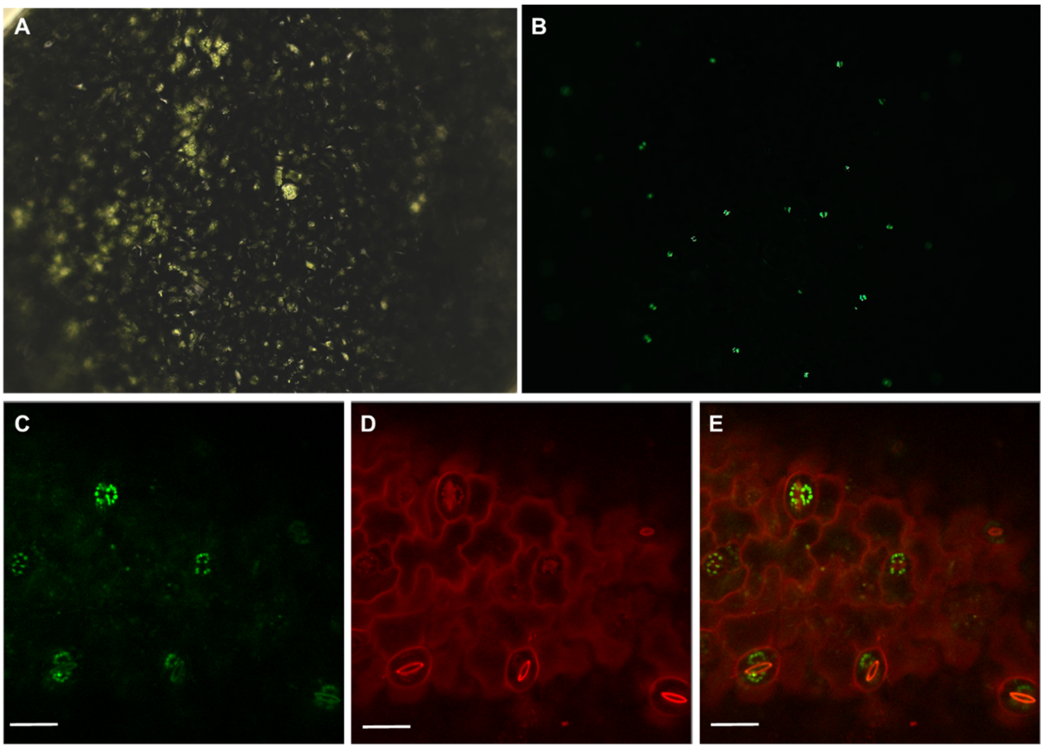

Figure S5. APx-R-YFP signal is retained in guard cells chloroplasts. A, B) Fluorescence microscopy image of expanded cotyledon from AtAPx-R-YFP OE-2 seedling showing APx-R-YFP persistence in stomata, while depleted from surrounding cells. C-E) Confocal image of AtAPx-R-YFP OE-1 expanded cotyledon. In green, YFP fluorescence (B, C, and E). In red, propidium iodide fluorescence (D and E). Scale: 25 µm.

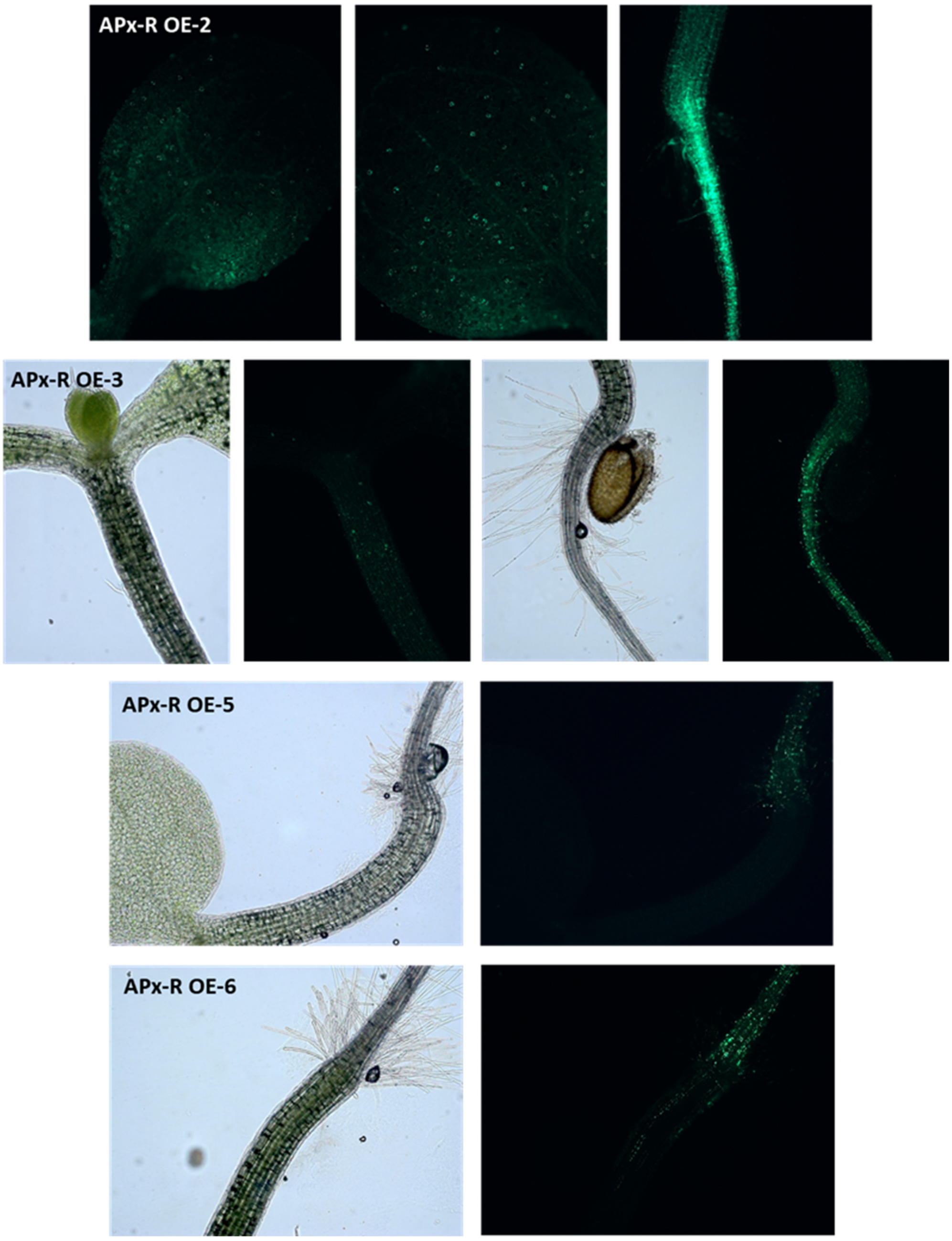

Figure S6. Recombinant AtAPx-R-YFP is detectable in roots but does not accumulate in green tissues. Fluorescence microscopy images obtained from cotyledons and roots of four-day-old AtAPx-R-YFP OE T2 seedlings from four independent lines (OE-2, OE-3, OE-5, and OE-6). YFP fluorescence is showed in green.

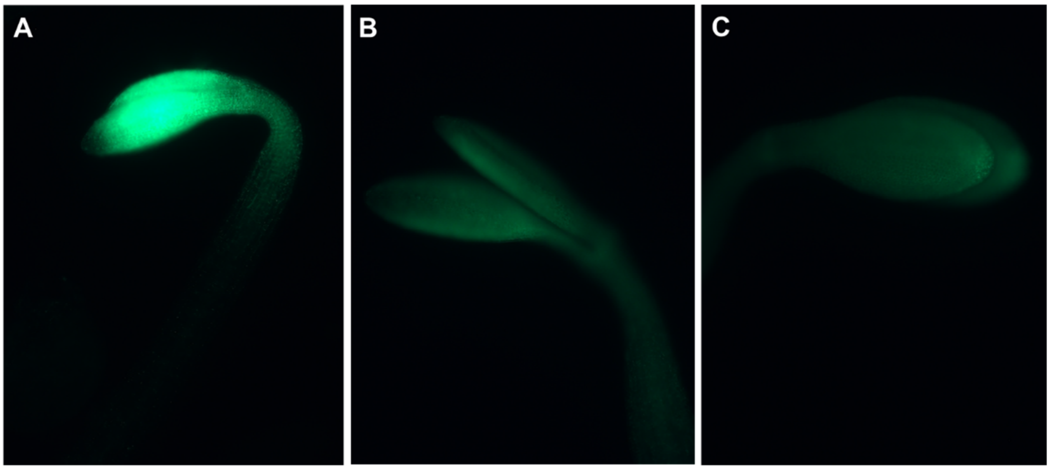

Figure S7. APx-R degradation is a proteasome-independent process. Four-day-old AtAPx-R-YFP OE-2 seedlings germinated in the dark and exposed to (A) 0 h of light, (B) 5 h of light, or (C) 5 h of light in the presence of 50 μM MG-132.

Table S1. Identification of Arabidopsis APx-R in proteomic data deposited on The Plant Proteome Database (http://ppdb.tc.cornell.edu/).

Experiment code	Description	Tissue	Sample	Genotype
1518	MS/MS analysis of plastoglobuli (PG) isolated from 6 weeks (in vegetative phase) or 12 weeks (flowering, advanced senescence stage) old rosettes of soil grown plants under short day light period (8h light / 16 h dark). This sample is from 6-week-old wt, purified PG replicate 2 (gel bands 31-36) Published in Bhuiyan et al. (2016) The Plant Cell 28(12):3020-3037	leaf (A. thaliana)	plastoglobuli	wild-type
1520	MS/MS analysis of plastoglobuli (PG) isolated from 6 weeks (in vegetative phase) or 12 weeks (flowering, advanced senescence stage) old rosettes of soil grown plants under short day light period (8h light / 16 h dark). This sample is from 6-week-old m48-1, purified PG replicate 2 (gel bands 43-48) Published in Bhuiyan et al. (2016) The Plant Cell 28(12):3020-3037	leaf (A. thaliana)	plastoglobuli	m48-1
1870	TAILS Arabidopsis thaliana (Col-0) baseline N-terminome, chloroplast stroma, dimethyl label (L), in solution digest and TAILS enrichment for N-termini (BioRep1), 3 samples, 4 LC/MS run following initial label, 7 LC/MS runs following TAILS. LTQ Orbitrap, 140 minutes gradient, 5 to 40% D solvent over 96 min, FTMS scans in Profile mode, normalized CE set to 30 (up from 25), min counts for MS/MS set to 1000, 2 MS/MS microscans summed. Data were searched against TAIR10 using MASCOT v2.4.0, semi-ArgC enzyme search specificity, 4 ppm and 0.8 Da MS and MS/MS mass tolerance respectively. Fixed modifications: dimethyl Lys, carboxamidomethyl Cys. Variable mods: dimethyl N-termini, acetyl N-termini, pyroGlu – Gln, oxidized Met.	leaf (A. thaliana)	stroma	wild-type
1875	TAILS Arabidopsis thaliana (Col-0) baseline N-terminome, total soluble leaf after dimethyl label (L), in solution/in gel digest with tryp, GluC or	leaf (A. thaliana)	total soluble	wild-type

	both and TAILS enrichment for N-termini determination (BioRep2), 6 samples, 7 LC/MS run following initial label, 20 LC/MS runs following TAILS, LTQ Orbitrap, 120 minutes gradient, 5 to 40% D solvent over 96 min, FTMS scans in Profile mode, normalized CE set 25, min counts for MS/MS set to 1000, 2 MS/MS microscans summed. Data were searched against TAIR10 using MASCOT v2.4.0, semi-ArgC enzyme search specificity, 4 ppm and 0.8 Da MS and MS/MS mass tolerance respectively. Fixed modifications: dimethyl Lys, carboxamidomethyl Cys. Variable mods: dimethyl N-termini, acetyl N-termini, pyroGlu – Gln, oxidized Met.			
1943	Arabidopsis stroma from Kenji for spectral counting, 1D SDS PAGE, blue coomassie stained, WT or CLPF mutant or ClpS1 mutant. This contributed to the publication Nishimura et al 2015 Plant Cell	leaf (A. thaliana)	stroma	wild-type
1945	Arabidopsis stroma from Kenji for spectral counting, 1D SDS PAGE, blue coomassie stained, WT or CLPF mutant or ClpS1 mutant. This contributed to the publication Nishimura et al 2015 Plant Cell	leaf (A. thaliana)	stroma	UVR
1946	Arabidopsis stroma from Kenji for spectral counting, 1D SDS PAGE, blue coomassie stained, WT or CLPF mutant or ClpS1 mutant. This contributed to the publication Nishimura et al 2015 Plant Cell	leaf (A. thaliana)	stroma	wild-type
1947	Arabidopsis stroma from Kenji for spectral counting, 1D SDS PAGE, blue coomassie stained, WT or CLPF mutant or ClpS1 mutant. This contributed to the publication Nishimura et al 2015 Plant Cell	leaf (A. thaliana)	stroma	clpS
2006	MS/MS analysis of plastoglobili (PG) isolated from wt Arabidopsis col-0, overexpressor M48 (OE) or RNAi M48 (RNAi) underexpression lines isolated from senescening rosettes of plants grown on soil for 35 days (flowering, advanced senescence stage) under long day light period (18h light / 6h dark). This sample is OE, purified PG replicate 2 (gel bands 13-24) Published in Bhuiyan et al. (2016) The Plant Cell 28(12):3020-3037	leaf (A. thaliana)	plastoglobuli	M48 overexpression
2008	MS/MS analysis of plastoglobili (PG) isolated from wt Arabidopsis col-0, overexpressor M48 (OE) or RNAi M48 (RNAi) underexpression lines isolated from senescening rosettes of plants grown on soil for 35 days (flowering, advanced senescence stage) under long day light period (18h light / 6h dark). This sample is RNAi, purified PG replicate 2 (gel bands 61-72) Published in Bhuiyan et al. (2016) The Plant Cell 28(12):3020-3037	leaf (A. thaliana)	plastoglobuli	M48 RNAi
2009	Analyses of prep1-1 x prep2-1, opda1-2 and Prep1-1 x prep2-1 x opda1-2 mutants for spectral counting by Jitae. Proteins (50µg per lane) are separated with a 1D SDS-PAGE, coomassie stained, extracted and digested with trypsin. Peptides from the digestion are analyzed by nano LC-MSMS on a Orbitrap-LTQ mass spectrometer	leaf (A. thaliana)	total leaf tissue	wild-type
2010	Analyses of prep1-1 x prep2-1, opda1-2 and Prep1-1 x prep2-1 x opda1-2 mutants for spectral counting by Jitae. Proteins (50µg per lane) are separated with a 1D SDS-PAGE, coomassie stained, extracted and digested with trypsin. Peptides from the digestion are analyzed by nano LC-MSMS on a Orbitrap-LTQ mass spectrometer	leaf (A. thaliana)	total leaf tissue	prep1-1xprep2-1
2012	Analyses of prep1-1 x prep2-1, opda1-2 and Prep1-1 x prep2-1 x opda1-2 mutants for spectral counting by Jitae. Proteins (50µg per lane) are	leaf (A. thaliana)	total leaf tissue	prep1-1xprep2-1xopda1-2

	separated with a 1D SDS-PAGE, coomassie stained, extracted and digested with trypsin. Peptides from the digestion are analyzed by nano LC-MSMS on a Orbitrap-LTQ mass spectrometer			
2014	Analyses of prep1-1 x prep2-1, opda1-2 and Prep1-1 x prep2-1 x opda1-2 mutants for spectral counting by Jitae. Proteins (50μg per lane) are separated with a 1D SDS-PAGE, coomassie stained, extracted and digested with trypsin. Peptides from the digestion are analyzed by nano LC-MSMS on a Orbitrap-LTQ mass spectrometer	leaf (A. thaliana)	total leaf tissue	prep1-1xprep2-1
2015	Analyses of prep1-1 x prep2-1, opda1-2 and Prep1-1 x prep2-1 x opda1-2 mutants for spectral counting by Jitae. Proteins (50μg per lane) are separated with a 1D SDS-PAGE, coomassie stained, extracted and digested with trypsin. Peptides from the digestion are analyzed by nano LC-MSMS on a Orbitrap-LTQ mass spectrometer	leaf (A. thaliana)	total leaf tissue	opda1-2
2020	Analyses of prep1-1 x prep2-1, opda1-2 and Prep1-1 x prep2-1 x opda1-2 mutants replica 3 for spectral counting by Jitae. Proteins (50μg per lane) are separated with a 1D SDS-PAGE, coomassie stained, extracted and digested with trypsin. Peptides from the digestion are analyzed by nano LC-MSMS on a Orbitrap-LTQ mass spectrometer	leaf (A. thaliana)	total leaf tissue	prep1-1xprep2-1xopda1-2
2031	TAILS experiment of chloroplast stroma to preform relative quantification (precursor-ion based) of protein N-termini between Col-0 control and icp55c-3 mutant, replicate 1A. WT - dimethyl +28, MU - heavy dimethyl +32, Light database search only. 100 micrograms protein of each genotype mixed after dimethyl labeling, proteins digested with trypsin and internal peptides crosslinked to HPG polymer then removed by ultrafiltration (30 kDa cutoff). LTQ Orbitrap, 140 minutes gradient, 5 to 40% D solvent over 96 min, FTMS scans in Profile mode, normalized CE set to 35 (up from 25), min counts for MS/MS set to 1000, 2 MS/MS microscans summed. Data were searched against TAIR10 using MASCOT v2.4.0, semi-ArgC enzyme search specificity, 4 ppm and 0.8 Da MS and MS/MS mass tolerance respectively. Fixed modifications: dimethyl Lys, carboxamidomethyl Cys. Variable mods: dimethyl N-termini, acetyl N-termini, pyroGlu – Gln, oxidized Met.	leaf (A. thaliana)	stroma	wild-type and icp55c-3
2032	TAILS experiment of chloroplast stroma to preform relative quantification (precursor-ion based) of protein N-termini between Col-0 control and icp55c-3 mutant, replicate 1A. WT - dimethyl +28, MU - heavy dimethyl +32, Heavy database search only. 100 micrograms protein of each genotype mixed after dimethyl labeling, proteins digested with trypsin and internal peptides crosslinked to HPG polymer then removed by ultrafiltration (30 kDa cutoff). LTQ Orbitrap, 140 minutes gradient, 5 to 40% D solvent over 96 min, FTMS scans in Profile mode, normalized CE set to 35 (up from 25), min counts for MS/MS set to 1000, 2 MS/MS microscans summed. Data were searched against TAIR10 using MASCOT v2.4.0, semi-ArgC enzyme search specificity, 4 ppm and 0.8 Da MS and MS/MS mass tolerance respectively. Fixed modifications: dimethyl Lys, carboxamidomethyl Cys. Variable mods: dimethyl N-termini, acetyl N-termini, pyroGlu – Gln, oxidized Met.	leaf (A. thaliana)	stroma	wild-type and icp55c-3
2033	TAILS experiment of chloroplast stroma to preform relative quantification (precursor-ion based) of protein N-termini between Col-0 control and icp55c-3 mutant, replicate 1B. MU - dimethyl +28, WT - heavy dimethyl +32, Light database search only. 100 micrograms protein	leaf (A. thaliana)	stroma	wild-type and icp55c-3

	of each genotype mixed after dimethyl labeling, proteins digested with trypsin and internal peptides crosslinked to HPG polymer then removed by ultrafiltration (30 kDa cutoff). LTQ Orbitrap, 140 minutes gradient, 5 to 40% D solvent over 96 min, FTMS scans in Profile mode, normalized CE set to 35 (up from 25), min counts for MS/MS set to 1000, 2 MS/MS microscans summed. Data were searched against TAIR10 using MASCOT v2.4.0, semi-ArgC enzyme search specificity, 4 ppm and 0.8 Da MS and MS/MS mass tolerance respectively. Fixed modifications: dimethyl Lys, carboxamidomethyl Cys. Variable mods: dimethyl N-termini, acetyl N-termini, pyroGlu – Gln, oxidized Met.			
2034	TAILS experiment of chloroplast stroma to preform relative quantification (precursor-ion based) of protein N-termini between Col-0 control and icp55c-3 mutant, replicate 1B. MU - dimethyl +28, WT - heavy dimethyl +32, Heavy database search only. 100 micrograms protein of each genotype mixed after dimethyl labeling, proteins digested with trypsin and internal peptides crosslinked to HPG polymer then removed by ultrafiltration (30 kDa cutoff). LTQ Orbitrap, 140 minutes gradient, 5 to 40% D solvent over 96 min, FTMS scans in Profile mode, normalized CE set to 35 (up from 25), min counts for MS/MS set to 1000, 2 MS/MS microscans summed. Data were searched against TAIR10 using MASCOT v2.4.0, semi-ArgC enzyme search specificity, 4 ppm and 0.8 Da MS and MS/MS mass tolerance respectively. Fixed modifications: dimethyl Lys, carboxamidomethyl Cys. Variable mods: dimethyl N-termini, acetyl N-termini, pyroGlu – Gln, oxidized Met.	leaf (A. thaliana)	stroma	wild-type and icp55c-3
2035	TAILS experiment of chloroplast stroma to preform relative quantification (precursor-ion based) of protein N-termini between Col-0 control and icp55c-3 mutant, replicate 2A. WT - dimethyl +28, MU - heavy dimethyl +32, Light database search only. 100 micrograms protein of each genotype mixed after dimethyl labeling, proteins digested with trypsin and internal peptides crosslinked to HPG polymer then removed by ultrafiltration (30 kDa cutoff). LTQ Orbitrap, 140 minutes gradient, 5 to 40% D solvent over 96 min, FTMS scans in Profile mode, normalized CE set to 35 (up from 25), min counts for MS/MS set to 1000, 2 MS/MS microscans summed. Data were searched against TAIR10 using MASCOT v2.4.0, semi-ArgC enzyme search specificity, 4 ppm and 0.8 Da MS and MS/MS mass tolerance respectively. Fixed modifications: dimethyl Lys, carboxamidomethyl Cys. Variable mods: dimethyl N-termini, acetyl N-termini, pyroGlu – Gln, oxidized Met.	leaf (A. thaliana)	stroma	wild-type and icp55c-3
2037	TAILS experiment of chloroplast stroma to preform relative quantification (precursor-ion based) of protein N-termini between Col-0 control and icp55c-3 mutant, replicate 2B. MU - dimethyl +28, WT - heavy dimethyl +32, Light database search only. 100 micrograms protein of each genotype mixed after dimethyl labeling, proteins digested with trypsin and internal peptides crosslinked to HPG polymer then removed by ultrafiltration (30 kDa cutoff). LTQ Orbitrap, 140 minutes gradient, 5 to 40% D solvent over 96 min, FTMS scans in Profile mode, normalized CE set to 35 (up from 25), min counts for MS/MS set to 1000, 2 MS/MS microscans summed. Data were searched against TAIR10 using MASCOT v2.4.0, semi-ArgC enzyme search specificity, 4 ppm and 0.8 Da MS and MS/MS mass tolerance respectively. Fixed modifications:	leaf (A. thaliana)	stroma	wild-type and icp55c-3

	dimethyl Lys, carboxamidomethyl Cys. Variable mods: dimethyl N-termini, acetyl N-termini, pyroGlu – Gln, oxidized Met.			
2038	TAILS experiment of chloroplast stroma to preform relative quantification (precursor-ion based) of protein N-termini between Col-0 control and icp55c-3 mutant, replicate 2B. MU - dimethyl +28, WT - heavy dimethyl +32, Heavy database search only. 100 micrograms protein of each genotype mixed after dimethyl labeling, proteins digested with trypsin and internal peptides crosslinked to HPG polymer then removed by ultrafiltration (30 kDa cutoff). LTQ Orbitrap, 140 minutes gradient, 5 to 40% D solvent over 96 min, FTMS scans in Profile mode, normalized CE set to 35 (up from 25), min counts for MS/MS set to 1000, 2 MS/MS microscans summed. Data were searched against TAIR10 using MASCOT v2.4.0, semi-ArgC enzyme search specificity, 4 ppm and 0.8 Da MS and MS/MS mass tolerance respectively. Fixed modifications: dimethyl Lys, carboxamidomethyl Cys. Variable mods: dimethyl N-termini, acetyl N-termini, pyroGlu – Gln, oxidized Met.	leaf (A. thaliana)	stroma	wild-type and icp55c-3
2042	TAILS experiment of chloroplast stroma to preform relative quantification (precursor-ion based) of protein N-termini between Col-0 control and icp55c-3 mutant, replicate 3B. MU - dimethyl +28, WT - heavy dimethyl +32, Heavy database search only. 100 micrograms protein of each genotype mixed after dimethyl labeling, proteins digested with trypsin and internal peptides crosslinked to HPG polymer then removed by ultrafiltration (30 kDa cutoff). LTQ Orbitrap, 140 minutes gradient, 5 to 40% D solvent over 96 min, FTMS scans in Profile mode, normalized CE set to 35 (up from 25), min counts for MS/MS set to 1000, 2 MS/MS microscans summed. Data were searched against TAIR10 using MASCOT v2.4.0, semi-ArgC enzyme search specificity, 4 ppm and 0.8 Da MS and MS/MS mass tolerance respectively. Fixed modifications: dimethyl Lys, carboxamidomethyl Cys. Variable mods: dimethyl N-termini, acetyl N-termini, pyroGlu – Gln, oxidized Met.	leaf (A. thaliana)	stroma	wild-type and icp55c-3
2049	TAILS experiment of total soluble leaf protein to preform relative quantification (precursor-ion based) of protein N-termini between Col-0 control and prep1xprep2xoop (aabbcc) mutant, replicate 2B. MU - dimethyl +28, WT - heavy dimethyl +32, Light database search only. 100 micrograms protein of each genotype mixed after dimethyl labeling, proteins digested with trypsin and internal peptides crosslinked to HPG polymer then removed by ultrafiltration (30 kDa cutoff). LTQ Orbitrap, 140 minutes gradient, 5 to 40% D solvent over 96 min, FTMS scans in Profile mode, normalized CE set to 35 (up from 25), min counts for MS/MS set to 1000, 2 MS/MS microscans summed. Data were searched against TAIR10 using MASCOT v2.4.0, semi-ArgC enzyme search specificity, 4 ppm and 0.8 Da MS and MS/MS mass tolerance respectively. Fixed modifications: dimethyl Lys, carboxamidomethyl Cys. Variable mods: dimethyl N-termini, acetyl N-termini, pyroGlu – Gln, oxidized Met.	leaf (A. thaliana)	total soluble	wild-type and prep1-1xprep2-1xoop
2054	TAILS experiment of total soluble leaf protein to preform relative quantification (precursor-ion based) of protein N-termini between Col-0 control and prep1xprep2xoop (aabbcc) mutant, replicate 3B. MU - dimethyl +28, WT - heavy dimethyl +32, Heavy database search only. 100 micrograms protein of each genotype mixed after dimethyl labeling, proteins digested with trypsin and internal peptides crosslinked to HPG	leaf (A. thaliana)	total soluble	wild-type and prep1-1xprep2-1xoop

	polymer then removed by ultrafiltration (30 kDa cutoff). LTQ Orbitrap, 140 minutes gradient, 5 to 40% D solvent over 96 min, FTMS scans in Profile mode, normalized CE set to 35 (up from 25), min counts for MS/MS set to 1000, 2 MS/MS microscans summed. Data were searched against TAIR10 using MASCOT v2.4.0, semi-ArgC enzyme search specificity, 4 ppm and 0.8 Da MS and MS/MS mass tolerance respectively. Fixed modifications: dimethyl Lys, carboxamidomethyl Cys. Variable mods: dimethyl N-termini, acetyl N-termini, pyroGlu – Gln, oxidized Met.			
2055	TAILS experiment of chloroplast stroma to preform relative quantification (precursor-ion based) of protein N-termini between Col-0 control and cgep mutant, replicate 1A. WT - dimethyl +28, MU - heavy dimethyl +32, Light database search only. 100 micrograms protein of each genotype mixed after dimethyl labeling, proteins digested with trypsin and internal peptides crosslinked to HPG polymer then removed by ultrafiltration (30 kDa cutoff). LTQ Orbitrap, 140 minutes gradient, 5 to 40% D solvent over 96 min, FTMS scans in Profile mode, normalized CE set to 35 (up from 25), min counts for MS/MS set to 1000, 2 MS/MS microscans summed. Data were searched against TAIR10 using MASCOT v2.4.0, semi-ArgC enzyme search specificity, 4 ppm and 0.8 Da MS and MS/MS mass tolerance respectively. Fixed modifications: dimethyl Lys, carboxamidomethyl Cys. Variable mods: dimethyl N-termini, acetyl N-termini, pyroGlu – Gln, oxidized Met.	leaf (A. thaliana)	stroma	wild-type and cgep
2084	TAILS experiment of chloroplast stroma to preform relative quantification (precursor-ion based) of protein N-termini between Col-0 control and clpt1xclpt2 mutant, replicate 3A. WT - dimethyl +28, MU - heavy dimethyl +32, Heavy database search only. 100 micrograms protein of each genotype mixed after dimethyl labeling, proteins digested with trypsin and internal peptides crosslinked to HPG polymer then removed by ultrafiltration (30 kDa cutoff). LTQ Orbitrap, 140 minutes gradient, 5 to 40% D solvent over 96 min, FTMS scans in Profile mode, normalized CE set to 30 (up from 25), min counts for MS/MS set to 1000, 2 MS/MS microscans summed. Data were searched against TAIR10 using MASCOT v2.4.0, semi-ArgC enzyme search specificity, 4 ppm and 0.8 Da MS and MS/MS mass tolerance respectively. Fixed modifications: dimethyl Lys, carboxamidomethyl Cys. Variable mods: dimethyl N-termini, acetyl N-termini, pyroGlu – Gln, oxidized Met.	leaf (A. thaliana)	total soluble	wild-type and clpt1xclpt2
2085	TAILS experiment of chloroplast stroma to preform relative quantification (precursor-ion based) of protein N-termini between Col-0 control and clpt1xclpt2 mutant, replicate 3B. MU - dimethyl +28, WT - heavy dimethyl +32, Light database search only. 100 micrograms protein of each genotype mixed after dimethyl labeling, proteins digested with trypsin and internal peptides crosslinked to HPG polymer then removed by ultrafiltration (30 kDa cutoff). LTQ Orbitrap, 140 minutes gradient, 5 to 40% D solvent over 96 min, FTMS scans in Profile mode, normalized CE set to 30 (up from 25), min counts for MS/MS set to 1000, 2 MS/MS microscans summed. Data were searched against TAIR10 using MASCOT v2.4.0, semi-ArgC enzyme search specificity, 4 ppm and 0.8 Da MS and MS/MS mass tolerance respectively. Fixed modifications: dimethyl Lys, carboxamidomethyl Cys. Variable mods: dimethyl N-termini, acetyl N-termini, pyroGlu – Gln, oxidized Met.	leaf (A. thaliana)	total soluble	wild-type and clpt1xclpt2

2312	Isolated thylakoids were run out on SDS-PAGE, cut in 7 consecutive slices/lanes, tryptic in-gel digestion, extraction of peptides, following by nanoLC-MSMS on a QExactive MS instrument. Genotype is wild-type. biological replicate 1	leaf (A. thaliana)	thylakoids thylakoid membrane	wild-type
2313	Isolated thylakoids were run out on SDS-PAGE, cut in 7 consecutive slices/lanes, tryptic in-gel digestion, extraction of peptides, following by nanoLC-MSMS on a QExactive MS instrument. Genotype is abc1k1. biological replicate 1	leaf (A. thaliana)	thylakoids thylakoid membrane	abc1k1
2315	Isolated thylakoids were run out on SDS-PAGE, cut in 7 consecutive slices/lanes, tryptic in-gel digestion, extraction of peptides, following by nanoLC-MSMS on a QExactive MS instrument. Genotype is ab1ck6. biological replicate 1	leaf (A. thaliana)	thylakoids thylakoid membrane	ab1ck6
2316	PG were extracted by sonication from isolated thylakoids, followed by density flotation centrifugation, collection and concentrations. Isolated PGs were run out on SDS-PAGE, cut in 7 consecutive slices/lanes, tryptic in-gel digestion, extraction of peptides, following by nanoLC-MSMS on a QExactive MS instrument. Genotype is wild-type. biological replicate 2.	leaf (A. thaliana)	plastoglobuli	wild-type
2318	PG were extracted by sonication from isolated thylakoids, followed by density flotation centrifugation, collection and concentrations. Isolated PGs were run out on SDS-PAGE, cut in 7 consecutive slices/lanes, tryptic in-gel digestion, extraction of peptides, following by nanoLC-MSMS on a QExactive MS instrument. Genotype is abc1k3. biological replicate 2.	leaf (A. thaliana)	plastoglobuli	abc1k3
2319	PG were extracted by sonication from isolated thylakoids, followed by density flotation centrifugation, collection and concentrations. Isolated PGs were run out on SDS-PAGE, cut in 7 consecutive slices/lanes, tryptic in-gel digestion, extraction of peptides, following by nanoLC-MSMS on a QExactive MS instrument. Genotype is abc1k6. biological replicate 2.	leaf (A. thaliana)	plastoglobuli	ab1ck6
2320	Isolated thylakoids were run out on SDS-PAGE, cut in 9 consecutive slices/lanes, tryptic in-gel digestion, extraction of peptides, following by nanoLC-MSMS on a QExactive MS instrument. Genotype = wild-type. biological replicate 2.	leaf (A. thaliana)	thylakoids thylakoid membrane	wild-type
2321	Isolated thylakoids were run out on SDS-PAGE, cut in 9 consecutive slices/lanes, tryptic in-gel digestion, extraction of peptides, following by nanoLC-MSMS on a QExactive MS instrument. Genotype is abc1k1. biological replicate 2.	leaf (A. thaliana)	thylakoids thylakoid membrane	ab1ck1
2324	Isolated thylakoids were run out on SDS-PAGE, cut in 9 consecutive slices/lanes, tryptic in-gel digestion, extraction of peptides, following by nanoLC-MSMS on a QExactive MS instrument. Genotype is ab1ck3. biological replicate 2.	leaf (A. thaliana)	thylakoids thylakoid membrane	abc1k3
2325	Isolated thylakoids were run out on SDS-PAGE, cut in 9 consecutive slices/lanes, tryptic in-gel digestion, extraction of peptides, following by nanoLC-MSMS on a QExactive MS instrument. Genotype is ab1ck6. biological replicate 2.	leaf (A. thaliana)	thylakoids thylakoid membrane	abc1k6
